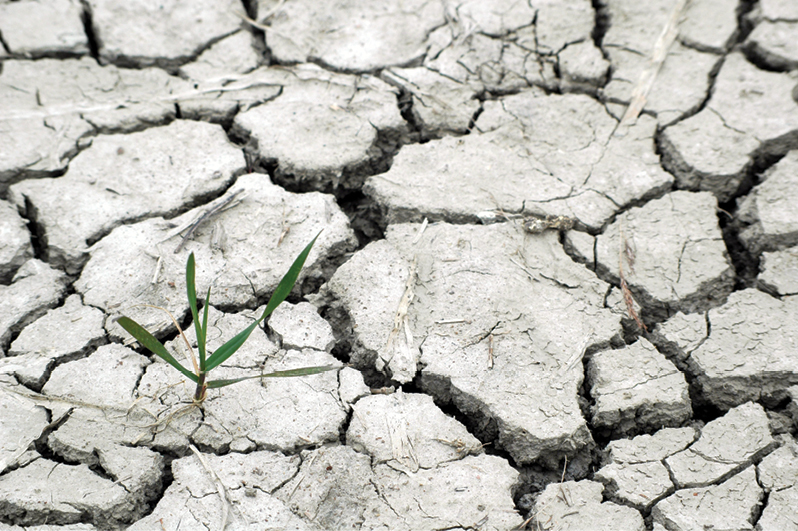# Improving crop performance under drought – cross-fertilization of disciplines

**DOI:** 10.1093/jxb/erx042

**Published:** 2017-02-18

**Authors:** Francois Tardieu, Rajeev K. Varshney, Roberto Tuberosa

**Affiliations:** 1 INRA LEPSE, 2 Place Viala 34060, Montpellier, France; 2 Research Programme – Genetic Gains, International Crops Research Institute for the Semi-Arid Tropics (ICRISAT), Patancheru-502 324, India; 3 Department of Agricultural Sciences, Viale Fanin 44, 40127 Bologna, Italy

**Keywords:** Climate change, crop productivity, drought tolerance, food security, water deficit.

**Better crop performance in dry environments is imperative for food security in the face of climate change. This has never been as true as in 2017, but the concern has existed for decades. The four InterDrought conferences held since 1995 have addressed issues associated with crop performance under drought with a clear multi-disciplinary approach. During this time *Journal of Experimental Botany* has been at the forefront in publishing the underlying experimental science encompassing the disciplines and scales of organization required in drought research. We hope that the papers highlighted here will be useful to, and instrumental for, broadening interdisciplinary understanding of drought tolerance.**

One of the most productive ways of tackling the agricultural challenge of drought is through cross-fertilization between areas of research, in particular crop physiology, agronomy, genetics, breeding, and environmental characterization/modelling. Scientists need to become familiar with multi-scale approaches from cells to crops subjected to water deficit, and this has been a major achievement of the InterDrought network (Box 1). Indeed, drought tolerance involves cellular aspects such as detoxification (Missihoun *et al.*, 2011) and osmotic adjustment (Blum, 2016), but also whole-plant signalling involved in the control of growth and transpiration under water deficit (Tardieu, 2016), the whole-plant control of shoot and root system architectures, and feedbacks between water capture, growth and transpiration at canopy level (Messina *et al.*, 2015). The exploitation of native genetic variability provides invaluable opportunities for improving plant performance based on mechanisms at any of these scales, namely cell, organ, whole-plant and canopy, in particular through progress in phenotyping (Fiorani and Schurr, 2013; see also the special issue ‘Phenotyping in plants’, introduced by Pieruschka and Lawson, 2015) and genomics-assisted breeding (Reynolds and Langridge, 2016).

**Box 1. InterDrought: 25 years of progress**

The link between crop performance and drought is now deeply embedded (Lobell *et al.*, 2008; Tester and Langridge, 2010) and highlighted in the last IPCC report (Field *et al.*, 2014). However, this wider appreciation by policymakers was being highlighted by scientists much earlier. InterDrought was created in 1992 with EEC funding, and at that time included European teams of researchers covering molecular biology, physiology, genetics and breeding. This quickly led on to the first international meeting for the network in Montpellier, in 1995 (Belhassen, 1997). Although the second InterDrought congress in 2005 was also in Europe (Rome) as the scope expanded so the meetings extended in 2009 to Shanghai, in 2013 to Perth and, in 2017, to Hyderabad.

Writing in the first contribution to a publication arising from InterDrought, Passioura (1997) noted that ‘*We can all tell that a cactus is more drought tolerant than a carnation. But when we look at crop plants, the features that confer drought tolerance are far from clear ... the traits we associate with xerophytes typically concern survival during drought, whereas with crops we are concerned with production – and insofaras the term “drought tolerance” has any useful meaning in an agricultural context, it must be defined in terms of yield in relation to a limiting water supply.*’ These words have been a clear ‘marker’ of InterDrought across its five congresses. We believe that the success of these meetings has enabled scientists from quite different disciplines to interact and acquire a broader view of the adaptive response of crops to such water scarcity. Image: Chickpea field trial in Patancheru, India, courtesy of Rajeev K. Varshney (Credit: L.Vidyasagar, ICRISAT).

**Figure:**
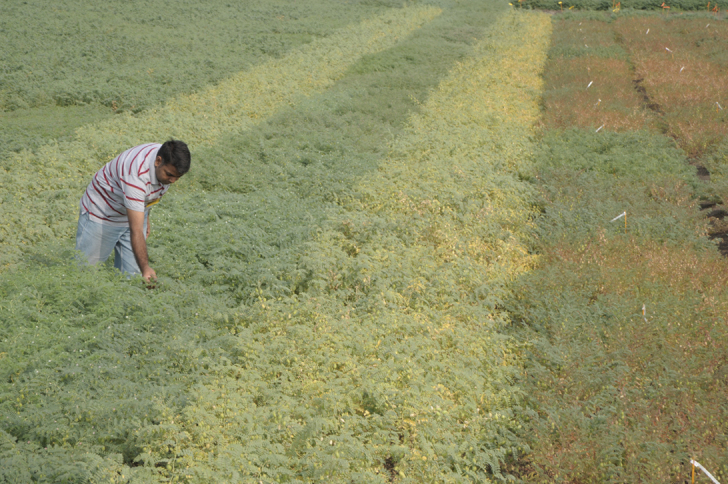

Improved knowledge of the physiological mechanisms involved in the control of transpiration and growth, and of their genetic make-up, paves the way for manipulating and eventually fine-tuning these controls in order to enhance their efficiency via genetic approaches (Habben *et al.*, 2014), possibly complemented by application of compounds that affect them (Park *et al.*, 2015). While agronomy is currently undergoing a major change in focus based on the widespread use of sensors, robots and imaging techniques resulting in precision agriculture, breeders are taking full advantage of our increasing ability to identify and tailor beneficial alleles able to enhance crop productivity and eventually mitigate the negative effects of drought.

## Rapid progress in molecular physiology and genomics applied to drought

Identification of the sources of tolerance and then cross-hybridization to recombine genomic segments form the basis of classical breeding for the development of drought-tolerant cultivars (Rauf *et al.*, 2016). However, the pace of development of improved drought-tolerant cultivars is slow, severely hampering the timely replacement of improved varieties for agriculture.

The controlling mechanisms for plant performance under drought are complex due to the multifaceted interplay between genetic components including genes, transcription factors, microRNAs (miRNAs), hormones, proteins, co-factors, ions and metabolites (reviewed by Janiak *et al.*, 2016; Tardieu, 2016). Advances in cost-effective sequencing and high-throughput genotyping technologies now mean that sequencing/genotyping large amounts of genetic material can be achieved within a limited period of time. These sequencing and genotyping data together with information from multi-environment phenotyping have enabled high-resolution genetic mapping leading to genetic dissection of improved yield under drought. Quantitative trait loci (QTLs) and candidate genes have been identified in various crops including rice (Zhou *et al.*, 2010), wheat (Maccaferri *et al.*, 2016), maize (Millet *et al.*, 2016) and chickpea (Jaganathan *et al.*, 2015; Kale *et al.*, 2015). Although gene editing has shown great potential as a powerful tool for improving any trait for which sequence variation is available (Bortesi *et al.*, 2015), this technology has yet to contribute to an appreciable improvement in drought tolerance in crop plants.

In addition to genetic studies conducted at the DNA level, efforts have also been made in candidate gene discovery through RNA deep sequencing (Chang *et al.*, 2016; Garg *et al.*, 2016; Shankar *et al.*, 2016) and microarray analysis (Mishra *et al.*, 2016). For example, whole-genome transcriptome-profiling studies have identified a large number of transcripts encoding members of various gene families reported to play an important role in abiotic stress tolerance, including *AP2/EREBP*, *bLHL*, *MYB*, and auxin-related families (Chang *et al.*, 2016; Garg *et al.*, 2016). Similarly, transcriptome analysis has identified the molecular mechanism underlying the high degree of plasticity of the water-deficit response in maize (Opitz *et al.*, 2016). Furthermore, induced expression of various transcription factors (e.g. MYB) have favourable effects under drought in maize (Casaretto *et al.*, 2016) and Arabidopsis (Scarpeci *et al.*, 2016).

Various studies have reported a role of miRNAs in abiotic stress tolerance (Shriram *et al.*, 2016), as in the case of drought-induced expression of *Hv*-miR827 in barley (Ferdous *et al.*, 2016). Likewise, up- or down-regulation of different miRNAs was found to be associated with improved performance in rice (Chung *et al.*, 2016) and barley (Hackenberg *et al.*, 2015) subjected to water deficit. Hormones also play an important role in the regulation of drought acclimation/adaptation ([Bibr CIT0008][Bibr CIT0063]; Visentin *et al.*, 2016). For instance, recent studies suggest a role for cytokinins in barley (Pospíšilová *et al.*, 2016), Arabidopsis (Nguyen *et al.*, 2016), tomato (Farber *et al.*, 2016) and rice (Talla *et al.*, 2016), and for strigolactones in tomato (Visentin *et al.*, 2016). These mechanisms have a crucial role for phenotypic plasticity. Indeed, the latter is a key trait for dealing with complex G×E interactions, as shown by Sadras *et al.* (2016), who explored the genetic control of phenotypic plasticity in chickpea. A systems-based approach which allows the integration of ‘omic’ technologies using computer-assisted theoretical and molecular biology would therefore help capture a global view of the complex mechanisms involved in the phenotypic plasticity associated with drought responses (Hussain *et al.*, 2016).

## Exploitation of genetic resources based on association mapping and genomic selection

Root features play a pivotal role in crop performance under water deficit, as well as in optimizing use of the available water resources (Messina *et al.*, 2015). In the past, roots have received limited experimental attention due to difficulties in phenotyping, particularly under field conditions. However, recent technical advances in root phenotyping and the utilization of high-throughput platforms have led to the publication of an impressive number of papers.

Particular attention has been devoted to the characterization of root mutants and the dissection of the genetic make-up governing root system architecture (RSA) and its effects on crop performance under different water regimes. The work of Jiang *et al.* (2016) highlights the role of strigolactones in the hormonal landscape that shapes RSA through the modulation of lateral root development via a tight interplay with auxins and cytokinins. Additional physiological work in Arabidopsis by Kircher and Schopfe (2016) supports the existence of periodic priming signals influencing lateral root formation along the growing root (see also the Insight article by Scheres and Laskowski, 2016). A valuable example of how to leverage molecular knowledge on lateral root growth to enhance the field performance of a drought-stressed crop is presented by Li *et al.* (2016). In rice, overexpression of transcription factor gene *MORE ROOT* (TaMOR) from wheat results in more roots and higher grain yield. TaMOR, a plant-specific transcription factor belonging to the ASYMMETRIC LEAVES2/LATERAL ORGAN BOUNDARIES (AS2/LOB) protein family, is highly conserved in wheat and its wild relatives. Notably, ITaMOR-D-overexpressing lines had larger root systems in Arabidopsis and rice, and produced a higher grain yield per plant. Therefore, TaMOR offers an opportunity to improve root architecture and increase yield in crops.

Among crops, cereals have a particularly complex and plastic root system whose components play different adaptive roles according to the growth stage and prevailing soil conditions (Hochholdinger, 2016). In maize, the QTL study of Gao and Lynch (2016) shows that a reduced crown root number is associated with greater root depth and improved water acquisition from drying soil. Previous studies have shown that major QTLs for RSA influence yield in maize grown under different water regimes (Landi *et al.*, 2007, 2010). Similar results have also been reported in rice (Price *et al.*, 2002; Uga *et al.*, 2011) and chickpea (Varshney *et al.*, 2013).

From an agronomic standpoint, the work of Lilley and Kirkegaard (2016) shows the importance of the time–space interplay between root depth and water capture as related to soil depth and annual resetting of soil water. This modelling study shows that capturing more water from deeper soil layers is not always the best option. Additionally, the simulation shows a greater impact of earlier sowing than modified root systems on water uptake, indicating that crop sequence must be managed tactically to optimize overall system benefits.

## ‘Whole-plant mechanisms’ which affect yield may differ between environments

Causal relationships between potential mechanisms and plant behaviour under drought are often far from straightforward or unidirectional due to multiple feedbacks at different timescales (Tardieu and Parent, 2017). For instance, it is well-known that early-flowering genotypes tend to escape drought compared with later genotypes, because flowering time and physiological maturity occur earlier in the season when the soil water reserve is not depleted. This is at the expense of potential biomass accumulation because of a shorter period in which photosynthesis can occur over the plant’s life cyle. Kazan and Lyons (2016) show that evolution may well have resulted in elaborate mechanisms in Arabidopsis plants subjected to water deficit which fine-tune the escape strategy and avoid its negative trade-offs. In particular earliness is controlled differentially in the case of drought in late or early-maturing accessions via differential expression of genes involved in the floral transition. This results in interesting feedback loops between floral transition, water uptake and growth. Another interesting example arises from the study of Christopher *et al.* (2016) (see also the Insight article by Rebetzke *et al.*, 2016*b*). Stay-green is often considered as a trait *per se* that confers drought tolerance. Christopher *et al*. considered the relationship between yield and different stay-green traits in eight contrasting environments. They found marked differences in relationships in well-watered conditions, and in water deficit occurring during either flowering or grain filling. Hence, indicators of stay-green have an effect on yield that is dependent on context. This may well be a general case for most traits involved in drought tolerance (Tardieu, 2012).

Similarly, there has been a long-standing debate about the effects of awns on yield, particularly under water deficit. Rebetzke *et al.* (2016*a*) propose a Solomon-like judgment: the presence or absence of awns has opposite effects on grain number and grain size, resulting in no major effect on yield (see also the Insight article by Guo and Schnurbusch, 2016). Indeed, the allocation of assimilates to awns decreases floret fertility, but favours grain filling particularly in dry environments. However, the respective effects on grain number and grain yield may have amplitudes that are context-dependent.

Hence, Blum (2016) states that drought tolerance needs to be re-defined in a better way by distinguishing the environment as sensed by plants (stress), the early plant reactions (strain) that trigger signalling processes and, eventually, acclimation processes. It is noteworthy that evolutionary geneticists usually reserve the term ‘adaptation’ for the selection of these acclimation processes over generations. As stated by Maron *et al.* (2016), fine-tuning concepts has large consequences for annotating so-called stress-resistance genes with markedly different effects in time and scales of organization (Tardieu and Parent, 2017). The latter considerations may considerably complicate early screening for drought tolerance based on root and shoot (Avramova *et al.,* 2016).

**Box 2. *Journal of Experimental Botany* and research on environmental change**

*Journal of Experimental Botany* (*JXB*) has had a long association with research on global environmental change, including drought and food security, not least through the influence of Bill Davies, Editor in Chief from 1995 to 2007 (see Dodd *et al.*, 2015). The *JXB* publications which followed on from the InterDrought conferences over the past decade chart our developing understanding of crop responses: InterDrought II (Araus *et al.*, 2007); InterDrought III (Boyer, 2010); and InterDrought IV (Tuberosa *et al.*, 2014).

A number of related special issues of *JXB* have also recently been produced: ‘Roots to global food security’ (Dodd *et al.*, 2015); ‘Plant roots: new challenges in a changing world’ (Price, 2016); ‘From inspiration to impact: delivering value from global root research’ (Rebetzke, 2016), with links made between research, breeding and environmental challenges (e.g. see Reyes *et al.*, 2015); and ‘Breeding plants to cope with future climate change’ (Halford and Foyer, 2015), which includes consideration of food security planning (Halford and Foyer, 2015; McKersie, 2015). Image: Pixabay, CC0 Public Domain.

**Figure:**